# Enhanced cognitive function and antidepressant-like effects after krill oil supplementation in rats

**DOI:** 10.1186/1476-511X-12-6

**Published:** 2013-01-25

**Authors:** Karin Wibrand, Kjetil Berge, Michaël Messaoudi, Anaïs Duffaud, Debabrata Panja, Clive R Bramham, Lena Burri

**Affiliations:** 1 Department of Biomedicine and KG Jebsen Centre for Research on Neuropsychiatric Disorders, University of Bergen, Jonas Lies vei 91, Bergen, NO-5009, Norway; 2 Aker Biomarine ASA, Fjordalleen 16, Oslo NO-0115, Norway; 3 ETAP-Applied Ethology Department of Neuropsychopharmacology, 13 rue Bois de la Champelle, Vandoeuvre-lès-Nancy, 54500, France

**Keywords:** BDNF, Cognition, Depression, Imipramine, Krill oil, Omega-3, Fatty acids, Phospholipids

## Abstract

**Background:**

The purpose of the study was to evaluate the effects of krill oil (KO) on cognition and depression-like behaviour in rats.

**Methods:**

Cognition was assessed using the Aversive Light Stimulus Avoidance Test (ALSAT). The Unavoidable Aversive Light Stimulus (UALST) and the Forced Swimming Test (FST) were used to evaluate the antidepressant-like effects of KO. Imipramine (IMIP) was used as the antidepressant reference substance.

**Results:**

After 7 weeks of KO intake, both males and females treated with KO were significantly better in discriminating between the active and the inactive levers in the ALSAT from day 1 of training (p<0.01). Both KO and IMIP prevented resignation/depression on the third day in the UALST. Similarly, a shorter immobility time was observed for the KO and IMIP groups compared to the control in the FST (p<0.001). These data support a robust antidepressant-like potential and beneficial cognitive effect of KO. Changes in expression of synaptic plasticity-related genes in the prefrontal cortex and hippocampus were also investigated. mRNA for brain-derived neurotrophic factor (*Bdnf*) was specifically upregulated in the hippocampus of female rats receiving 7 weeks of KO supplementation (p=0.04) and a similar trend was observed in males (p=0.08). Males also exhibited an increase in prefrontal cortex expression of *Arc* mRNA, a key protein in long-term synaptic plasticity (p=0.05). IMIP induced clear effects on several plasticity related genes including *Bdnf* and *Arc.*

**Conclusions:**

These results indicate that active components (eicosapentaenoic acid, docosahexaenoic acid and astaxanthin) in KO facilitate learning processes and provide antidepressant-like effects. Our findings also suggest that KO might work through different physiological mechanisms than IMIP.

## Background

Dietary intake of n-3 polyunsaturated fatty acids (PUFAs) is known to be beneficial for general health [1]. It is well documented that supplementation of the bioactive n-3 PUFAs, eicosapentaenoic acid (EPA) and docosahexaenoic acid (DHA), positively influence the cardiovascular system [2] and show anti-inflammatory effects [3,4]. The brain contains up to 60% lipids and a large portion of these are PUFAs, the most abundant being DHA [5]. Clinical data and animal experiments have establish that n-3 PUFAs are involved in maintaining a healthy brain and enhancing brain functions such as memory and learning [6,7], reactivity, attention, cognitive performance, and mood [8]. The hippocampal formation is a brain region critical for learning and memory [9]. Major depression is associated with neuronal atrophy in the hippocampus and hippocampal volume is reduced in patients with depression [10,11].

Several epidemiological studies suggest that depression is associated with lower n-3 PUFA levels and that n-3 PUFA treatment can be used in the treatment and prevention of depressive disorders in both adult patients [12] and in children [13]. Recent animal studies have shown that dietary supplementation of n-3 PUFAs can reduce immobility in the Forced Swimming Test (FST), a test used for validating depression status, while deprivation of n-3 PUFA can promote aggression and depression [14-18]. One hypothesis is that n-3 PUFAs exert their effect on the nervous system by regulating cyclic AMP (cAMP), cAMP response element binding protein (CREB), and the expression of downstream target genes such as brain-derived neurotrophic factor (BDNF) [17,18]. This is in line with studies showing that expression of BDNF and activation of the BDNF receptor, is reduced in the hippocampus and neocortex of rodents subjected to behavioural stress but enhanced following treatment with antidepressant drugs [19-25].

Antarctic krill, *Euphausia superba,* is a shrimp-like zooplankton and constitutes one of the largest biomasses of an individual species. Both fish oil (FO) and krill oil (KO) contain high levels of the essential polyunsaturated lipids EPA and DHA, but bond in different structural forms. In FO, EPA and DHA are found in the form of triglycerides, whereas in KO they are mainly in the form of phospholipids [26]. In addition to high levels of phospholipids, KO contains the natural antioxidant astaxanthin, that protects the unsaturated bonds from oxidation [27], shows antianxiety-like effects [28], and impacts on memory improvement in a common laboratory mouse model (BALB/c mice) [29]. The high content of phospholipids makes krill-derived products such as KO and krill powder interesting as a source of n-3 PUFAs. As the different structure of krill fatty acids may affect their incorporation into neuronal cell membranes, krill products may be particularly effective in modulating neuronal membrane function and signalling, including processes of synaptic transmission and plasticity. At present there is only one animal study addressing the effect of krill-derived phospholipids on brain function in rats [30]. The authors observed that spatial learning ability (for the radial arm maze task) and cell generation in the dentate gyrus was enhanced after KO administration. Here, we studied the effect of KO on cognition (learning acquisition and working memory) and depression, and examined a possible modulation of genes linked to synaptic plasticity, memory and adaptive brain function.

## Results

After 6 weeks of treatment administration, the cognitive and antidepressant effects of KO in male and female rats were monitored with two different models, the Conditioned Light Extinction Test (ALSAT and UALST procedures) and FST.

### Cognition and depression-like behaviour in the Conditioned Light Extinction Test (CLET)

#### Cognition: The cognitive effects of KO were assessed using the Aversive Light Stimulus Avoidance Test (ALSAT) procedure of the CLET

**Figure 1:**
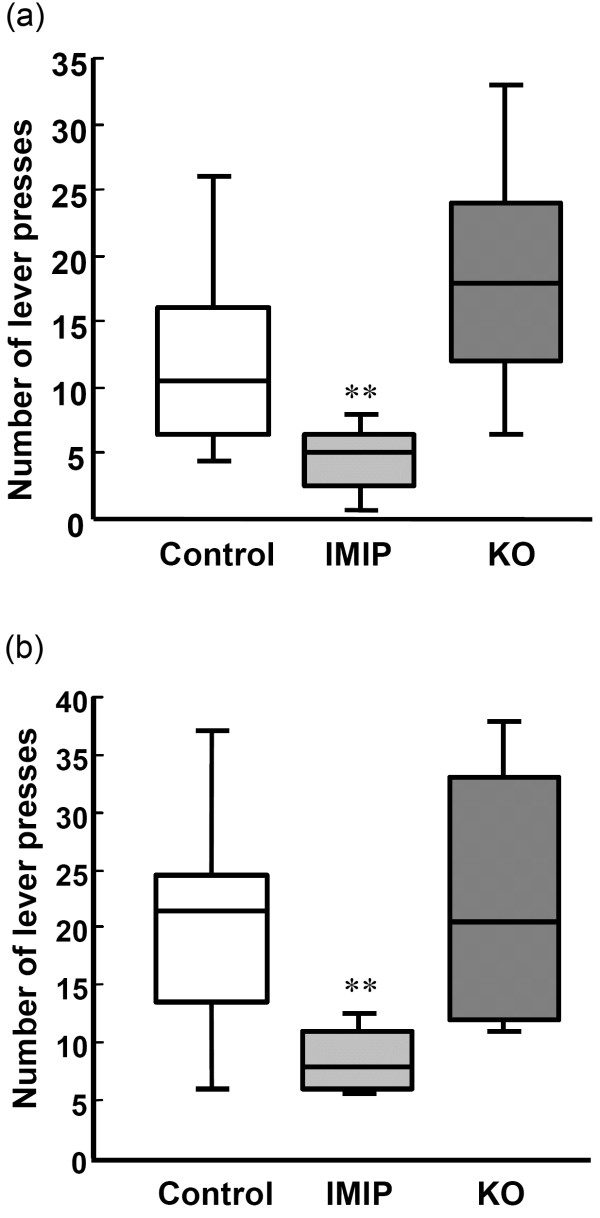
**The effect of KO and IMIP on total lever presses on day 1 in the Conditioned Light Extinction Test.** (**a**) Male rats and (**b**) Female rats. ** p<0.01 (vs. control). The box plots show the 10th, 25th 50th (median), 75th and 90th percentiles).

The rats were continuously exposed to the bright lit chamber unless an active bar-press occurred. The number of presses on the levers and the lever discrimination were measured during two 20 min learning acquisition sessions (Days 43 and 44) and in a third session (Day 45), when the active lever was inactivated. Total number of presses is a measurement of activity and alertness of the animals and a reduction in lever presses can be a sign of sedation. KO intake did not have any effect on the total number of presses (sum of active and inactive lever presses), whereas the antidepressant IMIP gave a significant reduction in total number of lever presses. The results from training day one (Day 43) are shown in Figure 1a and b.

The IMIP group had again less presses than the control group and the KO group did not change from the control (the total number of presses for all two days and both genders is presented in Additional file 1: Table S1).

**Figure 2:**
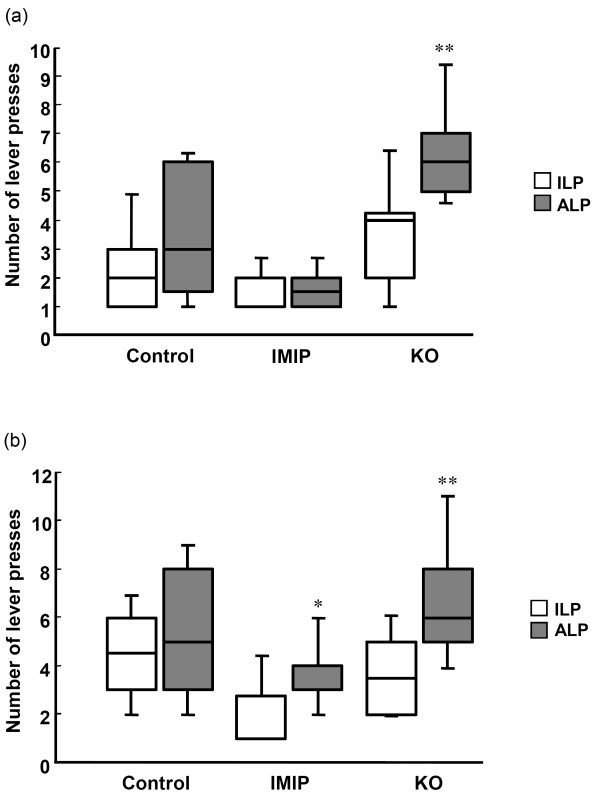
**The effect of KO and IMIP on active (ALP) and inactive (ILP) lever presses on day 1 in the Conditioned Light Extinction Test.** (**a**) Male rats. (**b**) Female rats. * p<0.05; ** p<0.01 (ALP vs. ILP). The box plots show the 10th, 25th 50th (median), 75th and 90th percentiles).

Although the total number of presses was similar in the control and KO groups, a clear difference in the ability to discriminate between the active and inactive lever (assessment of cognitive abilities, i.e. learning and working memory) was observed. Male and female rats receiving KO discriminated between the active lever and the inactive one during the whole two test sessions based on higher lever presses on the active lever during the light period and the discrimination was observed from the 1st day of training (Figure 2 and Additional file 1: Tables S2 and S3).

In addition, male rats administered IMIP showed a tendency for discrimination between the levers at day 2 and a significant successful discrimination was observed for female rats in day 1 and 2 (Additional file 1: Table S3). Day 1 is shown in Figure 2b).

#### Depression: the antidepressant-like effects of KO were evaluated in the Unavoidable Aversive Light Stimulus Test procedure (UALST) in the CLET

On day 3 (Day 45) the active lever was deactivated and the number of presses was recorded. A decrease in the number of presses on both levers and particularly on the active lever, when it was deactivated on day 3 compared to when it was active on day 2 can be interpreted as a sign of resignation or depression-like state, and as de-motivation due to the absence of positive reinforcement in the form of 30 s periods of darkness (Additional file 1: Table S4).

**Figure 3:**
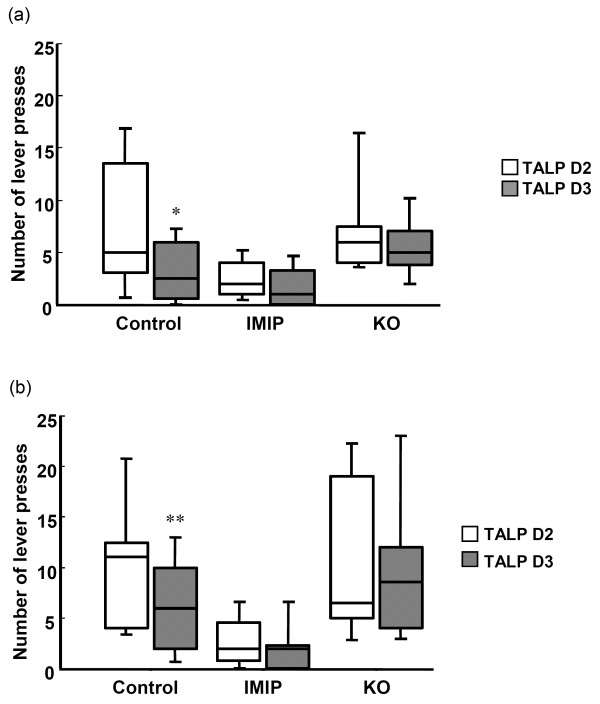
**The effect of KO and IMIP on resignation/depression in the Conditioned Light Extinction Test.** The total active lever presses on day 2 (TALP D2) vs. total active lever presses on day 3 (TALP D3). (**a**) Male rats (**b**) Female rats. * p<0.05 (TALP D2 vs. TALP D3). The box plots show the 10th, 25th 50th (median), 75th and 90th percentiles).

The total number of active lever presses (TALP) significantly decreased in control rats, while KO and IMIP prevented this effect (Figure 3 and Additional file 1: Table S4).

In addition, despite the absence of positive reinforcement, the KO groups of both genders continued to discriminate correctly between the two levers.

### The effect of KO in the Forced Swimming Test (FST)

**Figure 4:**
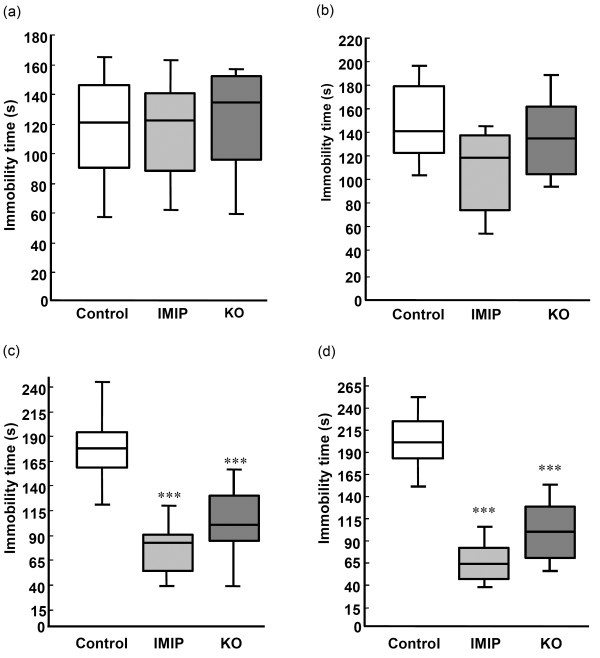
**Effects of KO and IMIP on immobility in the Forced Swimming Test.** The effect on immobility during the pre-test session in (**a**) Male rats and in (**b**) Female rats. The effect on immobility during the test session in (**c**) Male rats and in (**d**) Female rats. *** p<0.001 (vs. control). The box plots show the 10th, 25th 50th (median), 75th and 90th percentiles).

One way of measuring the state of depression in laboratory animals (rodents) is the FST. The immobility time (passive floating in water) is a measure of depression-like behaviour (learned helplessness) that can be modulated by antidepressant agents. After 7 weeks of treatment there was no difference in the immobility time between the three treatment groups when they were subjected to a pre-test session of the FST (Figure 4a and b and Additional file 1: Table S5: males: H_df(2)_=0.53; p=0.77; females: H_df(2)_=3.24; p=0.20). During the test session, significant differences were observed among immobility times in the male as in the female groups (H_df(2)_=18.86; p=0.0001 and H_df(2)_=22.44; p=0.0001, respectively). Decrease in the duration of immobility in groups receiving KO or IMIP compared to the control group were observed (males: KO: U=16.5; p=0.0005 and IMIP: U=2.50; p=0.0001; females: KO: U=4, p=0.0001 and IMIP: U=1; p=0.0004) (Figure 4c and d). When the immobility time was compared between the pre-test and the test session, control rats showed an increase in immobility (control: males: z=2.98, p=0.003 and females: z=3.06, p=0.002), while a decrease was observed for the KO (males: z=2.04, p=0.04 and females: z=2.92, p=0.004) and IMIP groups (males: z=2.70, p=0.007 and females: z=2.20, p=0.03) (Additional file 1: Table S6).

### The effect of KO on gene expression

**Figure 5:**
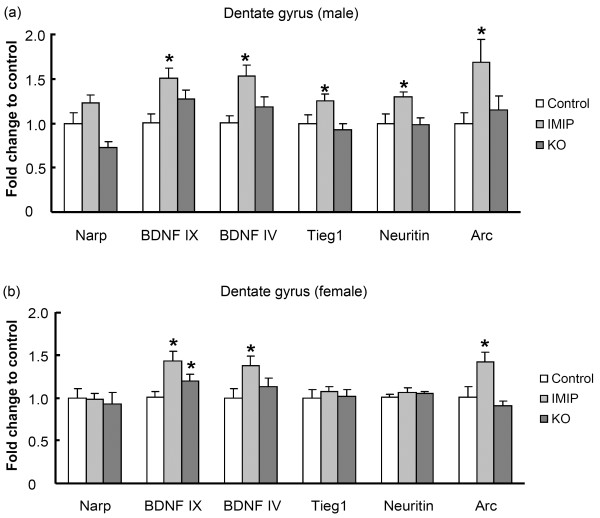
**Seven weeks of KO administration induces brain-derived neurotrophic factor (*Bndf*) mRNA expression in the hippocampus.** Changes in mRNA expression following KO administration is expressed as fold change relative to the control group. (**a**) Male rats n=12 control, n=12 IMIP, n=11-12 KO, (**b**) Female rats n=10-12 control, n=7-9 IMIP, n=13-14 KO. * p<0.05 from Student`s *t*-test.

**Figure 6:**
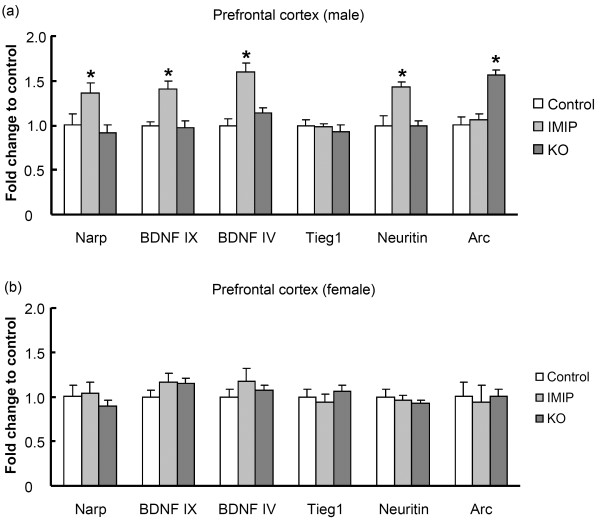
**Seven weeks of KO administration leads to gender-specific effects on activity-regulated cytoskeleton-associated** (***Arc*) mRNA regulation in the prefrontal cortex.** Changes in mRNA expression following KO administration is expressed as fold change relative to the control group. (**a**) Male rats n=12 control, n=11-12 IMIP, n=12 KO, (**b**) Female rats n=11-12 control, n=8-9 IMIP, n = 14 KO. * p<0.05 from Student`s *t*-test.

Quantitative real-time PCR was used to determine gene expression changes of *Bdnf* associated plasticity regulated genes in the prefrontal cortex and the dentate gyrus of the hippocampus. In the dentate gyrus (Figure 5a and b), the level of total *Bdnf* mRNA (primers targeting the common coding region, exon IX) was significantly up-regulated in females (p=0.04) by KO and a strong tendency towards up-regulation was observed in the male group (p=0.08) compared to their respective control groups. IMIP treatment resulted in an up-regulation in the dentate gyrus of both male and female rats of several plasticity-associated genes including total *Bdnf* (male p=0.004; female p=0.004), the activity-regulated *Bdnf* exon IV (male p=0.001; female p=0.03), and the activity-regulated cytoskeleton-associated protein (*Arc*) (male p=0.03; female p=0.04) (Figure 5a and b). In addition, male IMIP-treated rats exhibited significantly enhanced expression of *Neuritin* (p=0.02) and a tendency for increased expression of *Narp* and *Tieg1* (p=0.06) relative to control. In the prefrontal cortex (Figure 6a and b), *Arc* mRNA levels (p=0.05) were increased after KO administration in male, but not female rats. A trend towards increased expression of *Bdnf* exon VI was also observed in male, KO-treated rats. Increases in total *Bdnf* (p=0.01), *Bdnf* exon IV (p=0.0002), *Narp* (p=0.05) and *Neuritin* (p=0.015) was found in the prefrontal cortex of males receiving IMIP compared to the control group. None of the analyzed mRNAs differed significantly in females between KO and the control group.

## Discussion

The aim of this study was to evaluate the impact of oral administration of KO on depression, cognitive function and expression of genes linked to memory and changes in neuronal connectivity. The KO dose given to study rats is relevant for human consumption as the EPA and DHA amount given (1.25% KO of diet) corresponds to a human daily intake of 0.8 g EPA and DHA in an 8.4 MJ/day diet. A separate group that received the tricyclic antidepressant IMIP as a comparative positive control was included. The results showed that 7 weeks of KO supplementation significantly improved learning and working memory in the ALSAT and displayed significant antidepressant-like effects in the UALST and FST. In addition, KO enhanced expression of *Bdnf* mRNA, which is a gene implicated in neuronal growth and differentiation.

### Cognition and depression in the Conditioned Light Extinction Test (CLET)

In general the same effects were seen in both genders with the exceptions pointed out. The CLET is an ethological model used to score learning ability using the ALSAT procedure [31,32] and antidepressant-like effects using the UALST in rat [33].

The administration of KO did not result in any sign of sedation (similar number of total lever presses as in control group), a common problem with classic antidepressants and also seen in this study with IMIP. IMIP was previously shown to have a sedative effect on motor activity in rats [34]. KO administration increased the ability of the rats to discriminate between the active and the inactive lever, which can be interpreted as a sign of improved learning and memory function. A significantly higher number of presses were placed on the active rewarding lever compared to both control and IMIP receiving rats. Whereas the control group showed signs of resignation when the active lever was made inactive on test day 3, both IMIP and KO contributed to an antidepressant-like effect and no reduction in the number of lever presses between day 2 and day 3 was observed despite the absence of positive reinforcement.

Wistar Unilever rats are very sensitive to resignation/depression [35]. That is why these rats were chosen in this study to assess the antidepressant-like effects of KO, the primary objective of the study. With other laboratory rat strains, i.e. Wistar Han or Sprague–Dawley, generally rats learn to discriminate the active lever from the inactive one at the end of the first test session. In our study, after two-day training in the CLET, the KO and IMIP groups showed a higher number of active relative to inactive lever presses. However, the KO group displayed more active lever presses as early as day 1, at a time when the results with the control and IMIP groups did not show any discrimination. Although based on a single experiment, these results indicate that KO may accelerate learning processes. The light environment represents a stress for rats and is a disruptive factor in the acquisition of operant conditioning. Astaxanthin has anxiolytic-like effects [28] and is known to improve cognitive functions such as reaction time, attention and working memory [29,36]. The exact role of the astaxanthin in KO remains to be determined and if the rather low amounts given to KO-treated rats help to exploit without stress the environmental parameters of the learning situation in the CLET and to display better cognitive performances remains to be proven.

During the UALST session, when the active lever is deactivated, only KO rats displayed lever pressing activity relatively high compared with that of control and IMIP rats. Thus, KO rats did not resign themselves to suffer passively the unavoidable aversive light stimulus by continuing to press more on the levers and to discriminate the active lever from the inactive one. This effect could be mainly due to the n-3 PUFAs, astaxanthin having no known effect on depression [28].

### Depression in the FST

The antidepressant-like effect of KO was further studied using the FST. Dietary supplementation of KO resulted in reduced immobility time in water. This is consistent with studies demonstrating that alpha-linolenic acid (ALA) administration (ranging from 30 days to 15 weeks) and EPA and DHA administration (12 weeks) reduced immobility and climbing in the FST [14-18]. It is known that short-term administration of PUFAs fail to induce the antidepressant effects in FST [14,37].

Improved reference and spatial memory was recently reported in the radial maze test for rats that had received a diet supplemented with isolated phospholipids from KO [30]. Moreover, in these rats, DHA levels were shown to be significantly increased in the cerebral cortex, as well as in the hippocampus [30]. The KO used in the present study was previously shown to similarly increase brain DHA levels in Zucker rats [38]. This study was in comparison to FO, which did not lead to an increase in brain DHA levels. Underlying reasons explaining the difference between KO and FO administration might be related to different molecular forms of EPA and DHA (phospholipids *versus* triglycerides), which has recently been reviewed in more detail [39].

Taken together, these studies indicate that KO, which is rich in n-3 containing phospholipids, can increase DHA levels in the brain and may protect from depression-like behaviour, as measured by the UALST and FST. In addition, the study suggests that KO can improve learning acquisition and working memory as evaluated in the ALSAT.

Very recently, it was shown that dietary supplementation of KO attenuates inflammation and oxidative stress in ulcerative colitis in rats [40]. Especially, the role of inflammatory processes on emotion is indicated by findings of a link between depression and elevated systemic inflammatory markers [41]. It was demonstrated that antidepressant agents are able to prevent or suppress inflammation and depressive symptoms [42,43] induced by systemic injection of cytokines [44,45]. C-reactive protein (CRP), as a biomarker of inflammation [46], is involved in stroke and cognitive impairments [47], and KO phospholipids demonstrated anti-inflammatory responses, lowering CRP levels in human subjects [48,49]. Thus, KO might be used to influence mood and cognition in subjects with an inflammatory condition.

### Gene expression

Up-regulation of BDNF signalling and downstream gene expression is implicated in the action of several well-known antidepressant drugs [19,23,50]. N-3 PUFAs have also been shown to modulate BDNF expression and signaling in a brain region- and treatment-specific manner [18,51,52].

The present study shows that long-term KO administration to rats results in gender and brain region-specific changes in mRNA expression. Recent data also suggests that n-3 PUFAs can induce antidepressant effects associated with increased serotonergic neurotransmission and neurotrophin expression in the hippocampus [18,51,53].

Abnormalities in the PUFA content in the brain might affect mood control through effects on neurotrophin expression and signalling and deficiencies have been shown to reduce neurotrophin levels in the prefrontal cortex as well as in the hippocampus [54,55]. 6 and 15 weeks of ALA deprivation results in significantly decreased levels of BDNF expression, CREB transcription factor activity, and p38 mitogen-activated protein kinase (MAPK) activity in the frontal cortex [55].

Although IMIP and KO treatments both have antidepressant-like behavioural effects, these treatments do not have identical effects on gene expression. IMIP and KO both induce *Bdnf*, but only IMIP enhanced the expression of genes previously associated with BDNF signaling and long-term synaptic plasticity [19,56]. Hence, the antidepressant-like effects of IMIP and KO are correlated with distinct neurobiological mechanisms at the level of gene expression. However, it remains possible that KO regulates BDNF-responsive target genes on a different time course than does IMIP. The observed increase of *Bdnf* mRNA level observed in the present study might be a result of increased transcription or an effect on RNA turnover. Another study with n-3 PUFA supplementation (0.5% EPA and 1% DHA for 12 weeks) showed antidepressant effects, a change in CREB protein levels, but no increase in hippocampal BDNF protein levels [17]. This might be explained by differences in types, forms and amounts of n-3 PUFAs, and the fact that real-time PCR is a more sensitive method then Western blot. On the other hand, inclusion of 1.2% DHA in the diet was able to increase BDNF protein levels in rat hippocampal tissue after brain trauma [52].

Arc has key regulatory roles in protein synthesis-dependent synaptic plasticity, memory formation, and postnatal cortical development [57,58]. Interestingly, KO treatment induced *Arc* in the male prefrontal cortex, although *Bdnf* levels were not altered. On the other hand IMIP induced the expression of *Bdnf* and several BDNF-regulated genes, but did not increase *Arc* expression under these conditions. Another possible mechanism of *Arc* induction is through activation of muscarinic cholinergic receptors (mAchR). It has previously been shown *in vitro* and *in vivo* that activation of mAchR induces Arc expression [59-61].

In KO, the major phospholipid species is phosphatidylcholine, which may provide a source for acetylcholine biosynthesis [62]. Learning ability has previously been associated with increased cerebral levels of acetylcholine after the intake of a DHA supplemented diet in a rat model and different sources of DHA intake increase the release of acetylcholine from the rat hippocampus [63]. Future studies need to investigate possible changes in acetylcholine levels after KO administration.

## Conclusions

IMIP and KO both enhance cognition and provide antidepressant-like effects. IMIP and KO treatments are associated with enhanced *Bdnf* mRNA expression but have distinct effects on the expression of *Arc* and other synaptic-plasticity associated genes, suggesting partially distinct neurobiological mechanisms. Subject to the confirmation of these results in clinical trials, KO, with the synergy of its various components such as n-3 phospholipids and astaxanthin, might play a role in depression and cognition, and thus offer a novel approach to alleviate neurological and psychiatric disorders.

## Methods

### Animals and diet

The animal experiments have been carried out in the laboratories of ETAP-Applied Ethology (Vandoeuvre-lès-Nancy, France). The experiments were performed in accordance to the guidelines provided by the ASAB Ethical Committee for the use of animals in behavioural research (Animal Behavior 2006; 71:245–53) and by the Canadian Council on Animal Care. All experimental procedures were carried out according to French regulations (decree no. 87–848) and in compliance with the European Economic Community Directives (86/609/CEE and 2010/63/EU) on animal welfare. In total 38 male and 38 female adult Wistar-Unilever rats (Harlan Laboratories, The Netherlands), with starting weights of 175-200 g (males) and 125-150 g (females) have been used in this study. The six-week old male and female rats were maintained in two separate facilities and housed two per cage in a regulated environment (temperature 22±2°C; humidity 50±10%; 12 h inverted light–dark cycle). The rats were allowed free access to food and tap water. After 7 days of acclimatization the rats were randomly divided into three different treatment groups. KO, (Aker Biomarine Antarctic AS, Norway), was daily administered by oral gavage at a dose corresponding to 1.25% of the daily ration of food for 7 weeks. The average dose of KO given was 0.2 g/rat/day. As a positive reference substance the tricyclic antidepressant Imipramine (IMIP) (Sigma-Aldrich, France) was given to a separate treatment group, and the control animals received water by oral gavage. Each day, KO was mixed with natural spring water (Cristaline, France) at the concentration of 33% (v/v) and stirred until homogenization just before its oral administration. The diet compositions are reported in Table 1.

**Table 1:** Diet composition (g/100 g of diet)

	**Control**	**IMIP**	**KO**
Protein	16.40	16.40	16.37
Fat	4.00	4.00	4.20
Carbohydrate	48.50	48.50	48.40
**Fatty acids**			
Palmitic acid	0.50	0.50	0.69
Stearic acid	0.10	0.10	0.11
Oleic acid	0.70	0.70	0.87
Linoleic acid	2.00	2.00	2.02
Linolenic acid	0.10	0.10	0.10
EPA	0.00	0.00	0.15
DHA	0.00	0.00	0.07

*IMIP* Imipramine, *KO* krill oil.

The volume of administration (VA) was calculated for each rat according to its group treatment and the mean weight of food consumed the 2 or 3 previous days as follow:

VA = (Dose_KO_*Weight_fi_/Density_KO_)*3

VA: volume of administration

Dose_KO_: dose of treatment (0.0125)

Weight_fi_: mean weight of food intake consumed the 2 or 3 previous days

Density_KO_: density of KO (0.979)

**Figure 7:**
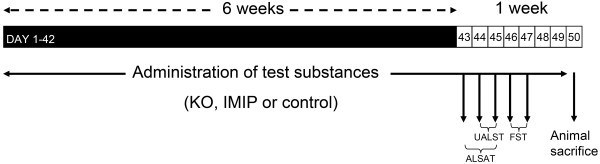
**The timing of test substance (KO, IMIP, or control) administration and behavioural studies.** The Conditioned Light Extinction Test (ALSAT: cognition, and UALST: depression) was performed on Days 43, 44 and 45 (corresponds to Day 1, 2 and 3), whereas the Forced Swimming Test (FST) was performed on Days 46 and 47. Animals were sacrificed at Day 50.

Imipramine was daily prepared fresh before its administration for 7 weeks. It was dissolved in water and then stirred until homogenization just before its oral administration at the daily dose of 20 mg/kg body weight, for 6 weeks before testing and 1 week during the testing period. The volume of administration was 7.5 ml/kg. The experimental set up is shown in Figure 7.

Because it is necessary to administer exact doses of the studied products by gavage, gastric feeding needles with a ball tip were used by experienced researcher. These needles were used to prevent introduction of the needle into the trachea and to prevent trauma to the oral cavity and to the esophagus. Feeding needles, fitted to a syringe, are carefully inserted through the mouth into the lower esophagus in hand restraint rats. Generally, introduction of the needle toward the rear of the mouth induces swallowing and the needle readily enters the esophagus to deliver the syringe content without inducing the stress associated with oral administration during the following treatments.

### Conditioned Light Extinction Test (CLET)

#### Cognition: the Aversive Light Stimulus Avoidance Test (ALSAT) procedure

The CLET apparatus (TESLA^®^, ETAP, France) consists of a chamber (50×40×37 cm) with a light level maintained at 1200 lux and allows the rats to escape from an aversive light stimulus by turning off the light. Two levers are included: an active one causing a 30-s period of darkness (positive reinforcement) whenever pressed down and an inactive one, which was not reinforced [31,32,64]. After 6 weeks of treatment administration, on the first and second day (Days 43 and 44) of the 7th week, each rat was continuously exposed to the brightly lit chamber during a 20 min learning acquisition session, unless an active barpress occurred (assessment of cognitive abilities, i.e. learning and working memory).

The total number of active and inactive lever presses was automatically recorded in both light and dark periods during the first and second test sessions (Days 43 and 44) and in light during the third session (Day 45). The lever pressing activity was studied through the total number of presses on both levers and the discrimination by comparing the active lever presses with the filtered inactive ones during the light period. Since each time the rat pressed the active lever a 30s period of darkness was induced, it was necessary to filter the inactive lever presses during the light period to be able to discriminate between the two levers during the two test sessions of learning. Thus, after pressing the inactive lever during the light period, the presses performed on this lever during the following 30s were not taken into account in assessing the discrimination between the levers.

#### Depression: Unavoidable Aversive Light Stimulus Test (UALST)

Following the learning sessions on days 43 and 44, on the third day (Day 45), the active lever was deactivated and the two levers were inactive during the 20 min-test session and thus the aversive light stimulus could not be avoided (assessment of the resignation behaviour i.e., depression-like state).

Resignation was measured by assessing the change of total lever presses on both levers and particularly on the active lever (total active lever presses: TALP) between the second session of learning acquisition (Day 44) and the third session (Day 45), where the active lever was deactivated and therefore where no positive reinforcement in the form of periods of darkness could be obtained. In this situation, extinction of the pressing behaviour is rapidly observed in untreated rats, while rats treated with antidepressant have been shown to continue to press on the levers [33]. Resignation or de-motivation can only be measured in a context in which positive reinforcement was experienced (active lever pressing). Therefore, only animals that were active in the first phase of the procedure (i.e. that pressed at least once each lever on Day 2) were included in the depression study.

### Forced Swimming Test (FST)

The FST apparatus (ETAP, France) consisted of a Plexiglas cylinder (50x20 cm i.d.) filled with water at 25°C to a depth of 30 cm. On day 46 (FST pre-test session), each rat was individually placed into the cylinder for 15 min. The water was changed after every trial to eliminate urine, faeces, and odor clues left from the previous rat. After the swimming session, the rat was removed from the cylinder, dried with towels, and then received its respective treatment. Then it was placed under a red light (30°C) for 20 min before returning it to its home cage. Twenty-four hours later (Day 47), the rat was once again individually subjected to the same swimming test for 5 min (FST test session). According to Porsolt’s procedure [65,66], with some modifications [35,67], in addition to the daily treatment of the previous weeks, between pre-test and test sessions, the treatments were administered orally three times as follows: immediately after the pre-test, as well as 5 h and 1 h before the test. Immobility, considered as the marker for depression, was defined as absence of movement, with the body inclined forward, passively floating, with immobile paws, as determined by video recordings.

The test was performed and immobility time scored by experimenters unaware of the administered products. The nature of the products was disclosed by the scientific director only after the end of the statistical data processing.

### Statistical analysis of behaviour data

The Kruskal-Wallis test (KWT) was used to compare the different treatment groups. When a significant difference was observed, the Mann-Whitney *U*-test (MWT) was performed to compare each group to the control group. For the repeated measures, the Wilcoxon test (WT) was used for each variable in each treatment group. The results are expressed as median with interquartile range values. Differences were considered to be significant at the level of P<0.05. All statistical analyses were carried out using the StatView^®^5 statistical package (SAS, Institute, Inc., USA).

### Biological sampling

On day 50, animals were anaesthetized with sodium pentobarbital (Ceva Santé Animale, Libourne, France) injected intraperitoneally at the dose of 100 mg/kg. Each rat was then euthanized by decapitation and trunk blood was collected in 2 EDTA tubes (Terumo, Leuven, Belgium) and immediately centrifuged (2200 g; 5 min; 4°C). Plasma was collected and aliquoted into 2×2 ml polypropylene tubes (Eppendorf, Le Pecq, France) and stored at −80°C for future use. Brains were removed from skulls immediately after decapitation and were cooled down during 1 min in a phosphate buffered solution (0.1 M; pH 7.4; ref: P7994; Sigma, St-Quentin Fallavier, France) at 4°C. Each brain was subsequently divided into 3 parts by realising 2 cuts in the coronal plane with a rat brain slicer matrix: a first cut was made at the level of the optic chiasm, and a second one between the cerebellum and cerebral hemispheres. The sections were then submerged in 15 ml of RNAlater^®^ reagent solution (Applied Biosystems, Courtaboeuf, France). After storage at 4°C overnight, samples were stored at −80°C until dissection and RNA purification.

### RNA isolation, cDNA synthesis and qPCR

Brains were maintained at −80°C until dissection. Dissections were carried out under RNAse free conditions. The pre-frontal cortex and hippocampal dentate gyrus were dissected and stored in RNAlater^®^ until RNA purification. Total RNA was purified with mirVana™ miRNA Isolation Kit (Ambion, Life technologies, Paisley, UK) according to the manufactures protocol. The tissues were homogenized in 600 μl ice cold lysis buffer provided with the mirVana kit, using the Prelyse system (Prelyse™ 24, Bertin Technologies, Montigny-le-Bretonneux, France) and recommended ceramic beads (03961ck14). The RNA was eluted in 100 μl 10 mM Tris–HCl and DNAse (Turbo DNAse Kit, Ambion) treated before cDNA synthesis. 300 ng of total RNA was used for cDNA synthesis using the iScript™ cDNA Synthesis Kit (BioRad, Hercules, CA, USA). The cDNA was diluted 10 times in RNAse and DNAse free water and analyzed with real time PCR on a Roche LightCycler 480. 10 μl reactions were run in 384-well plates using gene specific primers (Additional file 1: Table S7) and 2x PerfecCTa™ SYBR^®^ Green FastMix™ (Quanta BioScience, Inc., Gaithersburg, MD) at 60°C. A pooled sample of all cDNAs was used to generate standard curves for all genes analyzed. The standard curves were used for relative quantification of the individual samples.

NormFinder was used to check the stability of three different normalization genes [68]. In both the male and the female groups cyclophilin was clearly more stable than the other two and chosen for normalization. The GraphPad Software was used to remove outliers.

## Abbreviations

ALA: Alpha-linolenic acid; ALSAT: Aversive light stimulus test; ARC: Activity-regulated cytoskeleton-associated protein; BDNF: Brain-derived neurotrophic factor; cAMP: Cyclic adenosine monophosphate; CLET: Conditioned light extinction test; CREB: cAMP response element binding protein; CRP: C-reactive protein; DHA: Docosahexaenoic acid; EPA: Eicosapentaenoic acid; FST: Forced swimming test; FO: Fish oil; IMIP: Imipramine; KO: Krill oil; MAPK: Mitogen-activated protein kinase; KWT: Kruskal-Wallis test; MWT: Mann–Whitney *U*-test; PCR: Polymerase chain reaction; PUFA: Polyunsaturated fatty acids; TALP: Total number of active lever presses; TrkB: Tropomyosin receptor kinase B; UALST: Unavoidable aversive light stimulus test; WT: Wilcoxon test.

## Competing interests

The authors have read the journal's policy and have the following conflicts: Lena Burri and Kjetil Berge are employed in Aker BioMarine ASA, which partly paid for the study.

## Authors’ contributions

AD and MM have been in charge of the behavioural experiments. KW, DP and CB have been responsible for the planning and carrying out the gene expression analysis. LB and KB have designed the study. All authors have been contributing to the interpretation of the data and the writing of the manuscript. All authors read and approved the final manuscript.

## Supplementary Material

Additional file 1: Table S1 Effects of KO and IMIP on total lever presses on day 1, 2 in the ALSAT and day 3 in the UALST in male and female rats (Median with interquartile range values). **Table S2.** Effects of KO and IMIP on lever discrimination on days 1, 2 in the ALSAT, and day 3 in the UALST, in male rats (Median with interquartile range values). **Table S3.** Effects of KO and IMIP on lever discrimination on days 1, 2 in the ALSAT, and day 3 in the UALST, in female rats (Median with interquartile range values). **Table S4.** Effects of KO and IMIP on resignation/depression on day 3 in the UALST in male and female rats (Median with interquartile range values). **Table S5.** Effects of KO and IMIP on immobility during the pre-test session in the FST in male and female rats (Median with interquartile range values). **Table S6.** Effects of KO and IMIP on change in immobility time between the pre-test and test sessions in the Forced Swimming Test (Median with interquartile range values). **Table S7.** Primer sequences and accession numbers for analyzed genes. Click here for file
