# Supplementary material for: Enhanced cognitive function and antidepressant-like effects after krill oil supplementation in rats
Source: Lipids Health Dis. 2013 Jan 25;12:6. doi: 10.1186/1476-511X-12-6 (PMC3618203; doi:10.1186/1476-511X-12-6)
Supplement: Additional file 1: Table S1 — Effects of KO and IMIP on total lever presses on day 1, 2 in the ALSAT and day 3 in the UALST in male and female rats (Median with interquartile range values). Table S2. Effects of KO and IMIP on lever discrimination on days 1, 2 in the ALSAT, and day 3 in the UALST, in male rats (Median with interquartile range values). Table S3. Effects of KO and IMIP on lever discrimination on days 1, 2 in the ALSAT, and day 3 in the UALST, in female rats (Median with interquartile range values). Table S4. Effects of KO and IMIP on resignation/depression on day 3 in the UALST in male and female rats (Median with interquartile range values). Table S5. Effects of KO and IMIP on immobility during the pre-test session in the FST in male and female rats (Median with interquartile range values). Table S6. Effects of KO and IMIP on change in immobility time between the pre-test and test sessions in the Forced Swimming Test (Median with interquartile range values). Table S7. Primer sequences and accession numbers for analyzed genes. [file 1476-511X-12-6-S1.doc]

**SUPPLEMENTARY MATERIAL**

**Supplementary Table 1.**

**Primer sequences and accession numbers for analyzed genes**

| **Gene** | **Primer sequence** | **Acc number** |
| --- | --- | --- |
| *Arc* | Fw: CCCAGTCTGTGGCTTTTGTCA  Bw: GTGTCAGCCCCAGCTCAATC | NM_019361 |
| *BDNF* exon IX (amplifies all transcripts) | Fw: GCGGCAGATAAAAAGACTGC  Bw: GCAGCCTTCCTTCGTGTAAC | EF125690 |
| *BDNF* exon IV | Fw: CTGCCTTGATGTTTACTTTGAC  Bw: GCAACCGAAGTATGAAATAACC | EF125679 |
| *Cyclophilin A* | Fw: AGCACTGGGGAGAAAGGATT  Bw: GATGCCAGGACCTGTATGCT | BC059141 |
| *Narp* | Fw: GGCAAGATCAAGAAGACGTTG  Bw: TCCAGGTGATGCAGATATGGT | NM_001034199 |
| *Neuritin* | Fw: GGGACTTAAGTTGAACGGCA  Bw: ACCCAGCTTGAGCAAACAGT | NM_053346 |
| *Tieg1* | Fw: CATCTGTAGCCACCCAGGAT  Bw: TGGGACAGGCAAATTTCTTC | NM_031135 |

**Supplementary Table 2.** Effects of KO and IMIP on total lever presses on day 1, 2 in the ALSAT and day 3 in the UALST in male and female rats (Median with interquartile range values).

| **Sex** | **Parameter** | **Control**  **(n=12)** | **IMIP**  **(n=14)** | **KO**  **(n=14)** |
| --- | --- | --- | --- | --- |
| Male | Day 1  ALP+ILP | 10.50  (6.50-16.00) | 5.00  (2.5-6.50) | 18.00  (12.00-24.00) |
|  | M-W U-test  Significance* |  | U=22  p=0.004 | U=51.50  p=0.09 |
|  | Day 2  ALP+ILP | 13.50  (3.50-19.00) | 2.00  (0.50-6.00) | 12.00  (3.60-19.00) |
|  | M-W U-test  Significance* |  | U=26.50  p=0.008 | U=82.50  p=0.94 |
|  | Day 3†  ALP+ILP | 6.00  (1.50-10.50) | 0.50  (0.00-5.50) | 6.00  (4.00-9.00) |
|  | M-W U-test  Significance* |  | U=37  p=0.04 | U=73.50  p=0.59 |
|  | **Parameter** | **Control**  **(n=12)** | **IMIP**  **(n=12)**‡ | **KO**  **(n=14)** |
| Female | Day 1  ALP+ILP | 21.50  (13.50-24.50) | 8.00  (6.00-11.00) | 20.50  (12.00-33.00) |
|  | M-W U-test  Significance* |  | U=20.50  p=0.003 | U=80.00  p=0.84 |
|  | Day 2  ALP+ILP | 16.50  (12.50-24.50) | 2.00  (1.50-4.00) | 11.50  (6.00-31.00) |
|  | M-W U-test  Significance* |  | U=9.50  p=0.0003 | U=64.50  p=0.32 |
|  | Day 3†  ALP+ILP | 10.50  (3.50-16.50) | 2.50  (0.00-4.00) | 13.00  (7.00-16.00) |
|  | M-W U-test  Significance* |  | U=25  p=0.02 | U=70  p=0.47 |

ALP, Active lever presses; ILP, Inactive lever presses; M-W: Mann-Whitney U-test.
*Significance (*vs*. Control).

†On day 3 the active lever is deactivated.

‡Two female rats died before the third test day in the IMIP group.

**Supplementary Table 3.** Effects of KO and IMIP on lever discrimination on days 1, 2 in the ALSAT, and day 3 in the UALST, in male rats (Median with interquartile range values).

| **Parameter** | **Control**  **(n=12)**† | **IMIP (n=8-9)**† | **KO**  **(n=13)**† |
| --- | --- | --- | --- |
| Day 1  ALP | 3.00  (1.50-6.00) | 1.50  (1.00-2.00) | 6.00  (5.00-7.00) |
| Day 1  ILP | 2.00  (1.00-3.00) | 2.00  (1.00-2.00) | 4.00  (2.00-4.25) |
| Wilcoxon test  Significance* | z=1.44  p=0.15 | z=0.45  p=0.65 | z=2.69  p=0.007 |
| Day 2  ALP | 3.50  (2.50-5.00) | 1.00  (1.00-2.00) | 4.00  (3.00-5.25) |
| Day 2  ILP | 2.50  (1.00-4.50) | 1.00  (0.00-1.00) | 3.00  (1.75-3.25) |
| Wilcoxon test  Significance* | z=0.68  p=0.50 | z=1.84  p=0.07 | z=2.29  p=0.02 |
| Day 3  ALP | 2.50  (0.50-6.00) | 1.00  (0.00-3.25) | 5.00  (3.75-7.00) |
| Day 3  ILP | 2.50  (0.50-6.00) | 1.00  (0.00-2.25) | 1.00  (0.75-3.50) |
| Wilcoxon test  Significance* | z=0.36  p=0.72 | z=82  p=0.41 | z=2.88  p=0.004 |

ALP, Active lever presses; ILP, Inactive lever presses.

*Significance (ALP *vs.* ILP).

†Only animals that made lever presses on both levers on day 2 were included in the statistical analysis.

**Supplementary Table 4. Effects of KO and IMIP on lever discrimination on days 1, 2 in the ALSAT, and day 3 in the UALST, in female rats (Median with interquartile range values).**

| **Parameter** | **Control**  **(n=12)**† | **IMIP (n=9-11)**† | **KO**  **(n=14)**† |
| --- | --- | --- | --- |
| Day 1  ALP | 5.00  (3.00-8.00) | 4.00  (3.00-4.00) | 6.00  (5.00-8.00) |
| Day 1  ILP | 4.50  (3.00-6.00) | 1.00  (1.00-2.75) | 3.50  (2.00-5.00) |
| Wilcoxon test  Significance* | z=1.13  p=0.26 | z=2.17  p=0.03 | z=3.06  p=0.002 |
| Day 2  ALP | 5.50  (3.00-7.50) | 2.00  (1.00-3.75) | 4.00  (3.00-7.00) |
| Day 2  ILP | 4.00  (2.50-4.50) | 1.00  (0.00-1.75) | 3.50  (1.00-5.00) |
| Wilcoxon test  Significance* | z=2.43  p=0.015 | z=2.00  p=0.046 | z=1.87  p=0.061 |
| Day 3  ALP | 6.00  (2.00-10.00) | 2.00  (0.00-2.25) | 8.50  (4.00-12.00) |
| Day 3  ILP | 3.00  (2.00-7.00) | 1.00  (0.00-1.25) | 3.50  (1.00-7.00) |
| Wilcoxon test  Significance* | z=1.16  p=0.25 | z=0.97  p=0.33 | z=2.99  p=0.003 |

ALP, Active lever presses; ILP, Inactive lever presses.

* Significance (ALP *vs.* ILP).

† Only animals that made lever presses on both levers on day 2 were included in the statistical analysis.

**Supplementary Table 5.** Effects of KO and IMIP on resignation/depression on day 3 in the UALST in male and female rats (Median with interquartile range values).

| **Sex** | **Parameter** | **Control**  **(n=12)**† | **IMIP**  **(n=9)**† | **KO**  **(n=13)**† |
| --- | --- | --- | --- | --- |
| Male | TALP  (D2) | 5.00  (3.00-13.50) | 1.50  (0.50-3.50) | 5.50  (4.00-7.00) |
|  | TALP  (D3) | 2.50  (0.50-6.00) | 0.00  (0.00-3.00) | 4.50  (3.00-7.00) |
|  | Wilcoxon test  Significance* | z=2.30  p=0.02 | z=0.85  p=0.4 | z=1.07  p=0.28 |
|  | **Parameter** | **Control**  **(n=12)**† | **IMIP**  **(n=9)**† | **KO**  **(n=14)**† |
| Female | TALP  (D2) | 11.00  (4.00-12.50) | 2.00  (1.00-3.50) | 6.50  (5.00-19.00) |
|  | TALP  (D3) | 6.00  (2.00-10.00) | 2.00  (0.00-3.00) | 8.50  (4.00-12.00) |
|  | Wilcoxon test  Significance* | z=3.08  p=0.002 | z=0.77  p=0.44 | z=0.25  p=0.81 |

TALP, Total active lever presses.

*Significance (D2 *vs.* D3).

†Only rats that made lever presses on both levers on day 2 were included in the statistical analysis.

**Supplementary Table 6. Effects of KO and IMIP on immobility during the pre-test session in the FST in male and female rats (Median with interquartile range values).**

| **Sex** | **Parameter** | **Control**  **(n=12)** | **IMIP**  **(n=12)** | **KO**  **(n=14)** |
| --- | --- | --- | --- | --- |
| Male | Immobility time (s) | 121.00  (90.50-146.00) | 122.50  (89.00-141.00) | 135.00  (96.00-152.00) |
|  | M-W U-test  Significance* |  | U=70.50  p=0.93 | U=73.00  p=0.57 |
|  | **Parameter** | **Control**  **(n=12)** | **IMIP**  **(n=7)**† | **KO**  **(n=14)** |
| Female | Immobility time (s) | 141.00  (122.50-179.50) | 118.00  (74.25-137.50) | 135.00  (104.00-162.00) |
|  | M-W U-test  Significance* |  | U=23.00  p=0.11 | U=64.00  p=0.30 |

*Significance (*vs.* Control).

†Five animals unexpectedly died (drowning or exacerbated sensibility).

**Supplementary Table 7. Effects of KO and IMIP on change in immobility time between the pre-test and test sessions in the Forced Swimming Test (Median with interquartile range values).**

| **Sex** | **Parameter** | **Control**  **(n=12)** | **IMIP**  **(n=10)**† | **KO**  **(n=14)** |
| --- | --- | --- | --- | --- |
| Male | Immobility time (s)  (Pre-test session) | 121.00  (90.50-146.00) | 110.50  (85.00-131.00) | 135.00  (96.00-152.00) |
|  | Immobility time (s)  (Test session) | 177.50  (159.00-194.50) | 82.50  (52.00-91.00) | 101.00  (85.00-130.00) |
|  | M-W U-test  Significance* | z=2.98  p=0.003 | z=2.70  p=0.007 | z=2.04  p=0.041 |
|  | **Parameter** | **Control**  **(n=12)** | **IMIP**  **(n=7)**‡ | **KO**  **(n=14)** |
| Female | Immobility time (s)  (Pre-test session) | 141.00  (122.50-179.50) | 118.00  (74.25-137.50) | 135.00  (104.00-162.00) |
|  | Immobility time (s)  (Test session) | 201.50  (183.50-225.50) | 64.00  (46.75-81.75) | 100.00  (71.00-128.00) |
|  | M-W U-test  Significance* | z=3.06  p=0.002 | z=2.20  p=0.03 | z=2.92  p=0.004 |

*Significance (Pre-test *vs.* test).

†Two animals unexpectedly drowned during the test session.

‡Five animals unexpectedly died (drowning or exacerbated sensibility).
